# Bipolar Dislocation of the Proximal Phalanx of Toe: A Rare Injury

**DOI:** 10.7759/cureus.10490

**Published:** 2020-09-16

**Authors:** Kishore Vellingiri, Nagakumar J S, Shaikh Nazeer

**Affiliations:** 1 Orthopaedics, Sri Devaraj Urs Academy of Higher Education and Research, Kolar, IND

**Keywords:** floating proximal phalanx of toe

## Abstract

We report a very rare case of bipolar fracture with dislocation of the proximal phalanx or floating proximal phalanx of the toe. The literature has shown that closed reduction gives a lesser chance of success considering the inherent anatomy of the foot. We report a case of a 40-year-old male with an open injury to his right foot involving bipolar dislocation of the proximal phalanx of the third toe with extensor digitorum longus tendon injury and fracture of the neck of the fourth proximal phalanx of the right foot. With the realization that open reduction and Kirschner wire fixation are good options in patients with such a presentation, a prompt and accurate diagnosis with a treatment plan was made, which lead to clinical success.

## Introduction

Floating metatarsals are a rare and complex type of injury in the world of trauma to the foot. We report one of the very few cases of bipolar fracture-dislocation of the proximal phalanx or floating proximal phalanx of the toe, as described in the medical literature. The injury is typically found by concomitant dislocations of the metatarsals from both articular ends ("bipolar dislocations"). Irreducible dorsal dislocation of the interphalangeal joint of the great toe is rare [1,2]. Closed reduction gives a lower chance of success considering the inherent anatomy of the foot. We describe a case of bipolar dislocation of proximal phalanx of the toe, where closed reduction failed, resulting in the need for open reduction and Kirschner (K) wire fixation.

## Case presentation

A 40-year-old male patient was brought to R. L. Jalappa Hospital & Research Centre affiliated to Sri Devaraj Urs Medical College, Kolar, Karnataka, South India. The patient presented with an alleged history of road traffic accident, sustaining an open injury to his right foot. It involved the bipolar dislocation of the proximal phalanx of the third toe with extensor digitorum longus tendon injury and fracture of the neck of the fourth proximal phalanx of the right foot. Range of motion of the metatarsophalangeal joint of the third and fourth digits was painful and restricted. Active first, second, and fifth toe movements were present. No distal neurovascular deficits were noted. All other bones and joints were clinically normal. It was an isolated injury with no evidence of intracranial, thoracic, abdominal, or pelvic injury on clinical or radiographic examination. A focused assessment with sonography in trauma showed no signs of hemorrhage.

**Figure 1:**
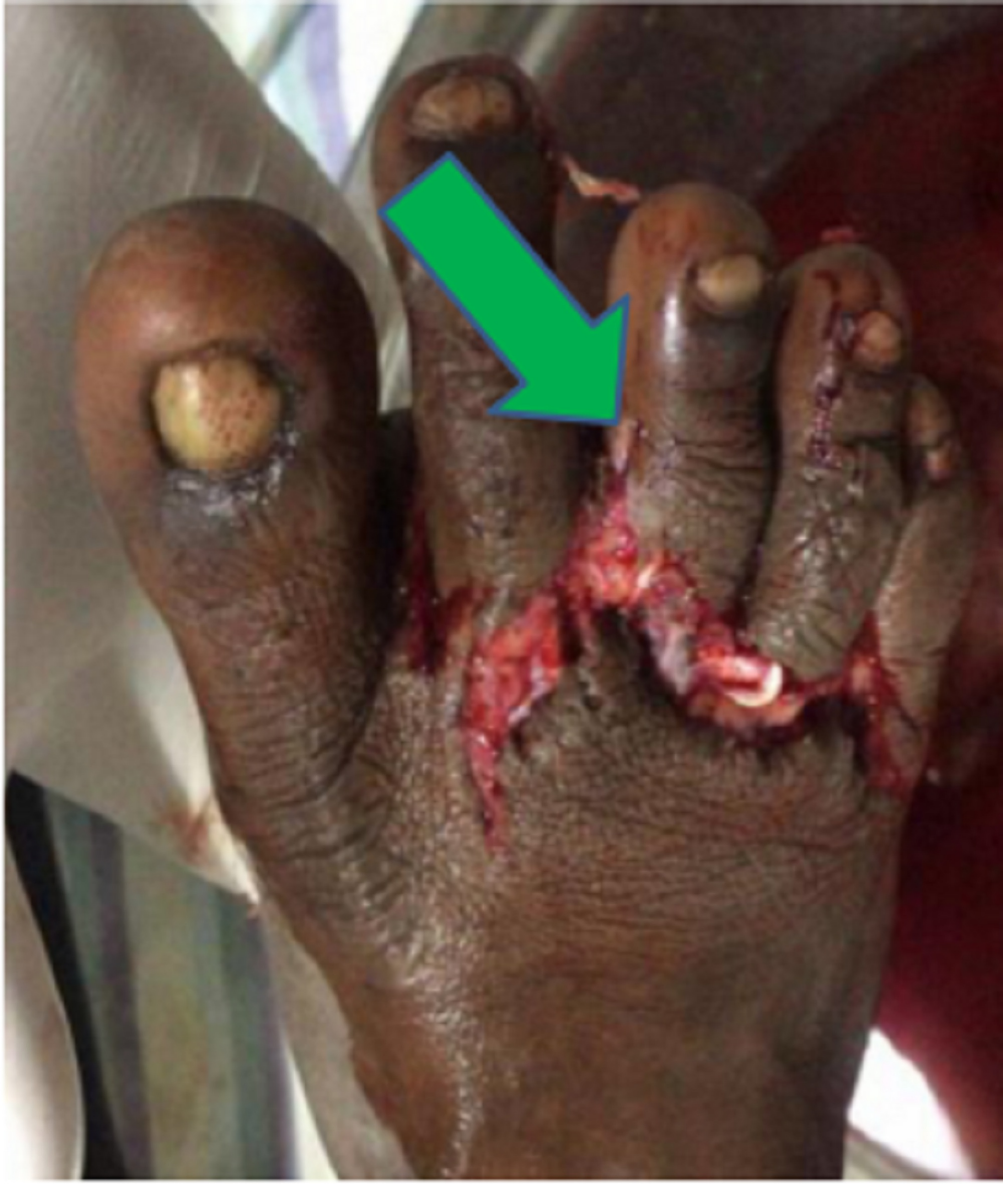
Clinical picture of the right foot following trauma.

**Figure 2:**
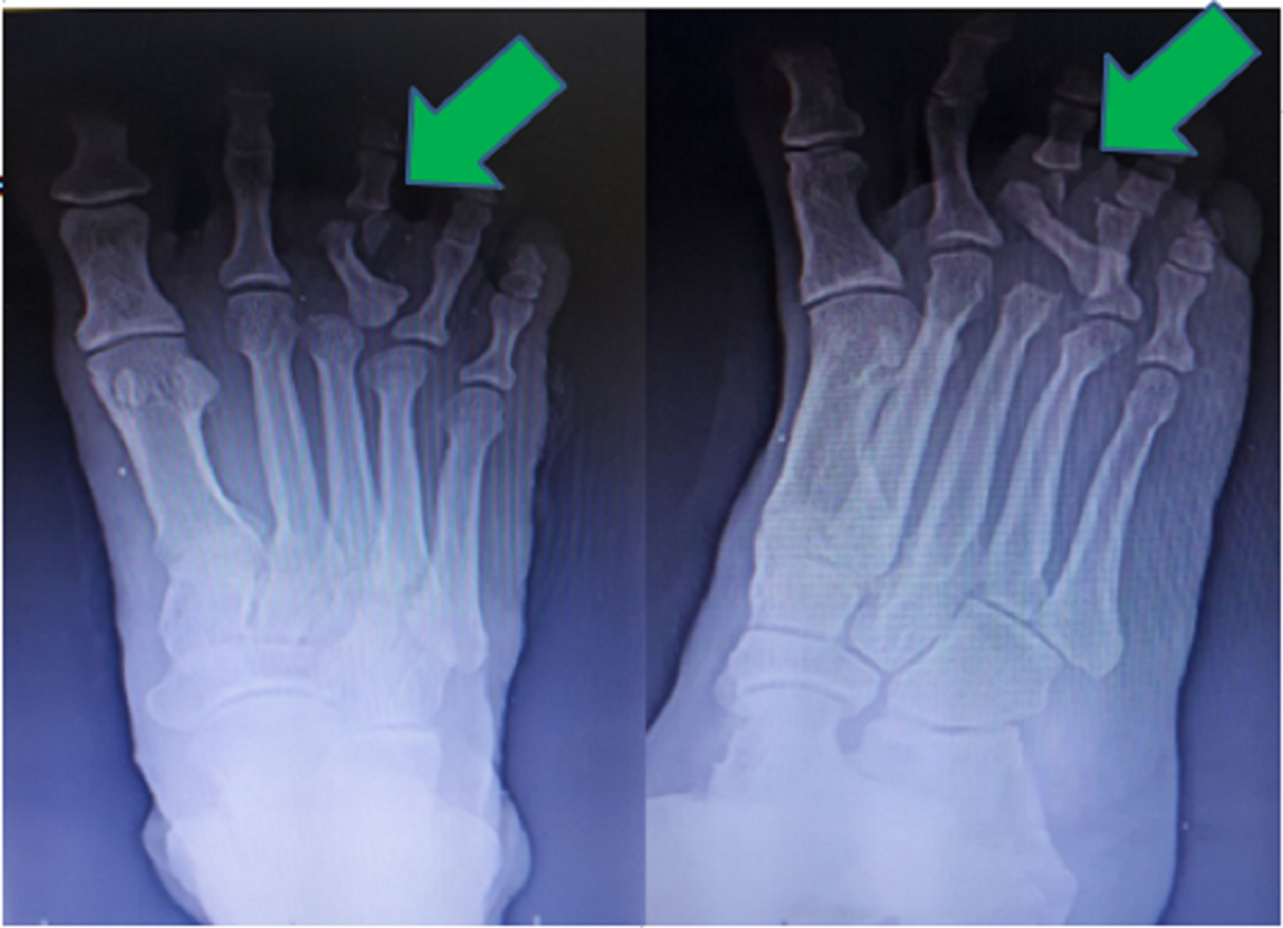
Plain radiographs of the right foot in anteroposterior and oblique views showing bipolar dislocation of the proximal phalanx of the third toe and neck of the fourth proximal phalanx fracture.

**Figure 3:**
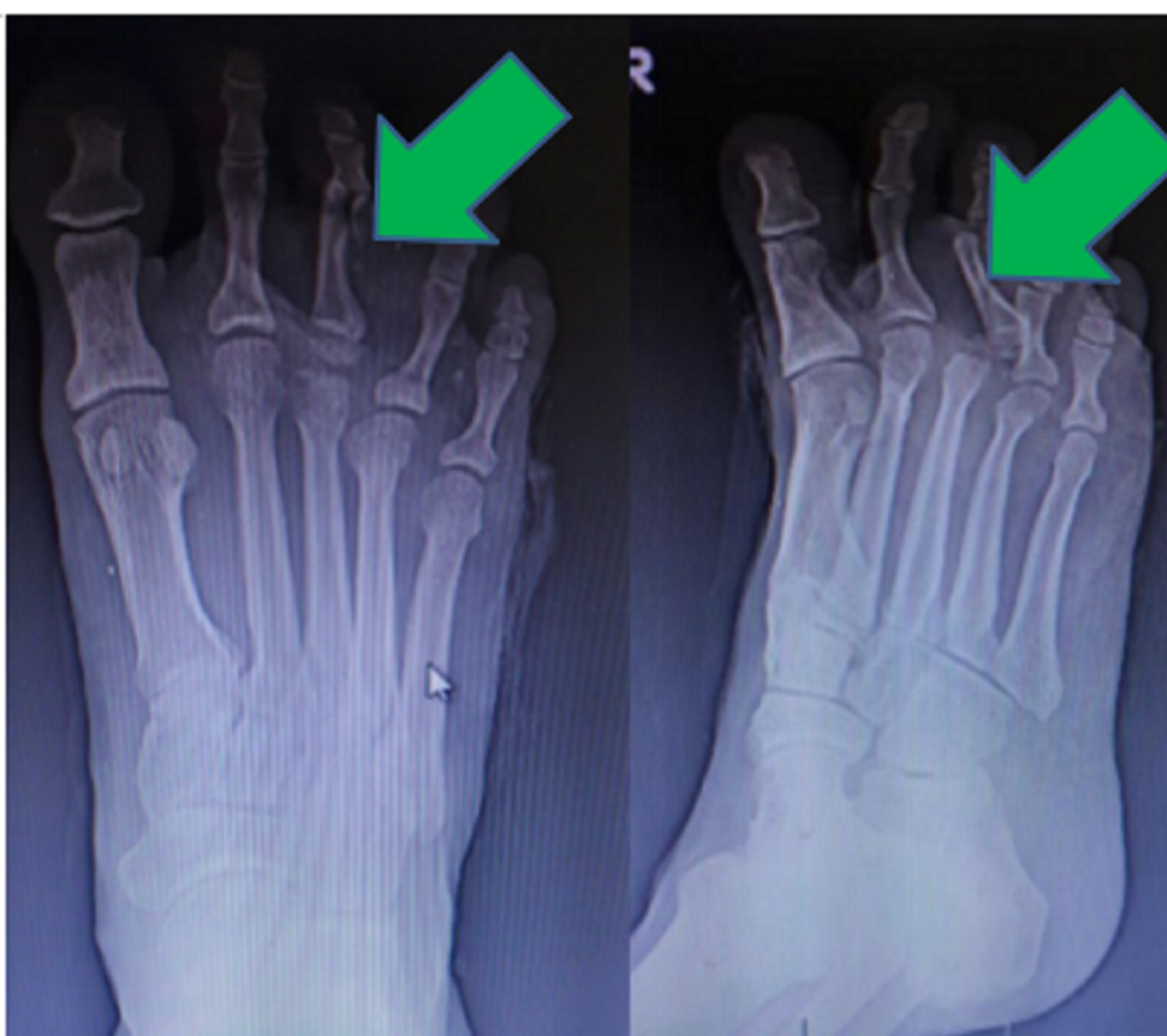
Failed closed reduction radiographs.

**Figure 4:**
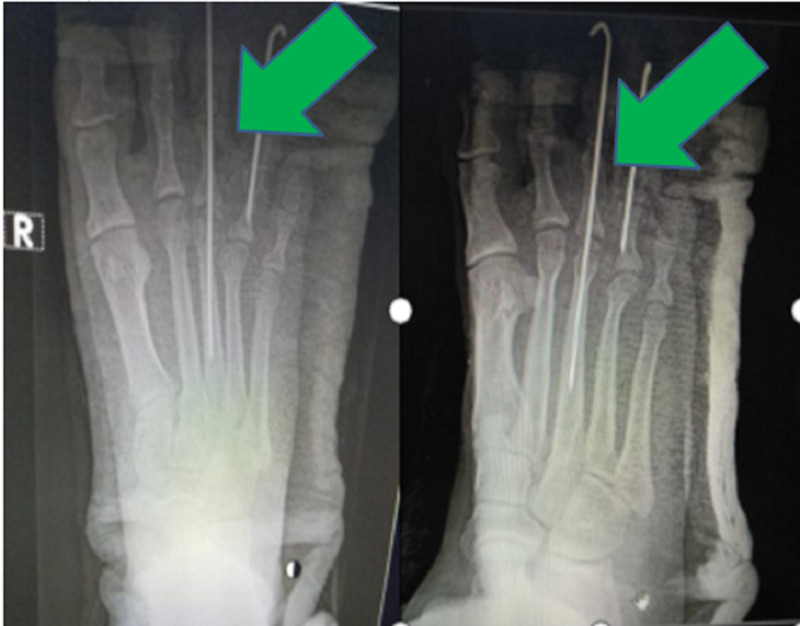
Post-reduction following open reduction with Kirschner wire fixation.

On arrival, tetanus prophylaxis was administered to the patient. Triple antibiotic prophylaxis consisting of amoxicillin-potassium clavulanate 1.2 g, amikacin sulfate 500 mg, and metronidazole 100 mL were administered in the emergency department. A brief bedside irrigation with 6 L of sterile saline was given, and the wound site was dressed with moist gauze. The patient was then provisionally stabilized with a short leg splint and sent for preoperative imaging. Clinical image and preoperative radiographs are provided in Figures 1, 2, respectively. After imaging was completed, a trial closed manipulation failed as expected, as shown in Figure 3. The patient was operated under spinal anesthesia. Three liters of sterile saline was then used to irrigate the wound. Gross wound contaminants were removed. The patient underwent wound debridement with extensor digitorum longus tendon repair of the fourth digit, and internal fixation with K-wire fixation for the third and fourth digit was performed. Post-operative image is shown in Figure 4. The post-operative period went uneventful. The superficial and deep wound infection never occurred at any point of time. This included physical signs (erythema, malodor, gross purulence, etc.) and hematological signs (rising of white blood cell count, erythrocyte sedimentation rate, C-reactive protein etc.). Post-operatively, the patient stayed in the hospital for seven days for wound monitoring. Daily physical and occupational therapy was routinely performed by the patient. The surgical site healed well. Active toe movements of the first to fifth digits were present.

Hospital course

Post-operatively, the patient received intravenous amoxicillin-potassium clavulanate 1.2 g twice daily for seven days, amikacin sulfate 500 mg twice daily for five days, and metronidazole 100 ml thrice daily for three days followed by oral amoxicillin-potassium clavulanate 625 mg twice daily for seven days. Analgesics and anti-edema measures were given. Protein-rich supplements along with personal hygiene measures were provided. A below-knee slab and strict non-weight bearing ambulation for six weeks were recommended. The K-wires were removed after six weeks, and partial weight-bearing was started with the help of a walker. Full weight-bearing was permitted after six months. The patient was being followed up at regular intervals. The patient returned to his work with no limitations on the activity or complications after his final follow-up.

## Discussion

English described a case of floating metatarsal in 1964 and defined the phenomenon of toe linked in the tarsometatarsal joint dislocations [3]. Leibner et al. in 1997 coined the term “floating’ metatarsals” [4]. Adequate primary treatment ensures satisfactory outcome. If undiagnosed, the end result is disorganization of the foot, leading to pain and incapacity. It is important to analyze the mechanism of injury and the treatment modalities.

Precise anatomical reduction of both joints is a crucial factor to achieve better function, as post-traumatic arthritis is directly proportional to the degree of damage to the articular surface. The bipolar dislocation is a permanent displacementsof a long bone or a flat bone from the two ends at the joints. This two-side dislocation occurs as a one-time injury or two-time injury [5]. The isolated bone is called a “floating bone” [6]. It is an exceptional injury usually published as case reports. An axial loading force resulting in dorsiflexion of the toes and equinus of the ankle is the typically attributed mechanism for the injury.

Unlike the floating first metatarsal, where there is a deformity of the foot or a cock-up deformity of the great toe, floating metatarsals of the lesser metatarsals may present only with gross swelling [7]. It is important to achieve reduction of the distal joint first in case of a first floating metatarsal, as it will aid in releasing tension over the plantar fascia and promoting easy proximal joint reduction [6]. For lesser metatarsals, reduction must be performed in the opposite order (proximal to distal) to release the tension of the dorsal interossei [8]. Regardless of the type of metatarsal involved, there appears to be a common consensus that the open approach is almost always required to achieve some or all reductions. For floating metatarsals, the order of reduction is probably more important than the type of fixation. It is important to have a close follow-up watching out for loss of reduction, implant failure, or development of arthritic changes in the mid-foot.

The challenge in closed reduction is likely to be a result of the interposed plantar capsule with the sesamoid. This has been described by Suwannahoy et al., wherein, with the dorsal approach, the extensor tendon may be retracted aside or split, with the latter giving better surgical exposure [9]. After open reduction of the interphalangeal joint, the joint displayed increased laxity. This arises from overstretching of the capsule and the collateral ligaments at the time of injury [10]. There have been some more methods of stabilization described in the literature, such as bulky dressing, buddy splinting, and immobilization in a small leg cast for four weeks [10]. Trinquier et al. suggest that the sequence of repair in these injuries should be started from metatarsophalangeal joint. After reduction of the metatarsophalangeal joint, the plantar fascia relaxes and allows the Lisfranc joint to be reduced [11].

Initially, an attempt should be made to close reduction, and if it fails, open reduction is mandatory. However, there are proponents of the immediate open reduction in order to allow anatomical reduction of metatarsophalangeal joins and to treat the intra-articular lesions (osteochondral fractures, loose body, and interposed soft tissue) [12]. The emergency physician should be educated regarding diagnosis and referral to a foot surgeon or orthopedician to avoid further complications. In our present case with bipolar dislocation of the proximal phalanx of the third toe, closed reduction failed as expected. Open reduction with K-wire fixation is a good option in patients presenting with this type of injury. Therefore, a prompt, accurate diagnosis with a treatment plan needs to be made involving the patient and the surgeon.

## Conclusions

In this rare case report of a floating toe (third digit), we highlight the importance of open reduction as close reduction usually fails and the need of internal fixation with K-wire fixation in achieving and maintaining reduction. Our hypothesis is that open reduction and internal fixation is indicated for floating toe. It is very difficult to prove with a larger sample size as it is the rarest variety of fracture occurrence. The patient in our case report had better functional and clinical outcomes following open reduction followed by internal fixation with K-wire fixation for the floating toe.

## Appendices

The abstract of the case was approved for presentation at the 50th Malaysian Orthopaedic Association Annual Scientific Meeting 2020 on June 24-26, 2021. The abstract is being scheduled to publish in the online supplement for the aforementioned conference journal SAOA combined Congress 2020. The conference in 2020 got postponed due to the COVID-19 pandemic.

A poster of the abstract is scheduled to be presented at the Virtual poster competition at the 50th Malaysian Orthopaedic Association Annual Scientific Meeting 2020 on June 24-26, 2021.
